# Dependency of the optical scattering properties of human milk on casein content and common sample preparation methods

**DOI:** 10.1117/1.JBO.25.4.045001

**Published:** 2020-04-11

**Authors:** Colin Veenstra, Dayna E. Every, Wilma Petersen, Johannes B. van Goudoever, Wiendelt Steenbergen, Nienke Bosschaart

**Affiliations:** a University of Twente, Technical Medical Centre, Faculty of Science and Technology, Biomedical Photonic Imaging Group, Enschede, The Netherlands; b Vrije Universiteit Emma Children’s Hospital, Dutch Human Milk Bank, Amsterdam University Medical Center, Amsterdam, The Netherlands

**Keywords:** homogenization, human milk, fat globules, casein, optical scattering

## Abstract

**Significance.** Quantifying human milk composition is important for daily nutritional management in neonatal intensive cares worldwide. Photonic solutions based on visible light can potentially aid in this analysis, as energy content of human milk depends largely on fat content, and the optical scattering properties of human milk predominantly depend on the size and concentration of fat globules. However, it is expected that human milk scattering changes upon homogenization, routinely done before analysis, which may affect fat globule size.

**Aim.** The first aim of this study was to investigate how the most common homogenization methods (gently inverting by hand, vortexing, and sonication) affect the optical properties of human milk. The second aim was to estimate the scattering contribution of casein micelles, the second most dominant scatterers in human milk.

**Approach.** We combined diffuse reflectance spectroscopy with spectroscopic optical coherence tomography to measure the scattering coefficient $\mu_{s}$, reduced scattering coefficient $\mu_{s}^{\prime }$, and anisotropy g between 450 and 600 nm.

**Results.** Sonication induced the strongest changes in $\mu_{s}$, $\mu_{s}^{\prime }$, and g compared to the gently inverted samples (203%, 202%, and 7%, respectively, at 550 nm), but also vortexing changed $\mu_{s}^{\prime }$ with 20%. Although casein micelles only showed a modest contribution to $\mu_{s}$ and g at 550 nm (7% and 1%, respectively), their contribution to $\mu_{s}^{\prime }$ was 29%.

**Conclusions.** The scattering properties of human milk strongly depend on the homogenization method that is employed, and gentle inversion should be the preferred method. The contribution of casein micelles was relatively small for $\mu_{s}$ and g but considerably larger for $\mu_{s}^{\prime }$.

## 1 Introduction

Human milk is the optimal source of nutrition for infants in early life.^1^^,^^2^ Breastfeeding supports infant survival, growth, health, and cognitive development^3^ and protects against many types of infections, diarrhea, and dental malocclusions.^1^ Also, mothers benefit from breastfeeding with a reduced risk for breast cancer, ovarian cancer, and improved birth spacing.^1^ As a consequence, it is estimated that the deaths of 823,000 children and 20,000 mothers can be prevented every year if all mothers worldwide would breastfeed their infants.^1^

Despite all these advantages, breastfeeding rates—especially in high-income countries—do not meet the advice of the World Health Organization for mothers to exclusively breastfeed their infants until the age of 6 months, followed by continued breastfeeding with complimentary foods until a minimum age of 2 years.^4^ Major reasons for mothers to stop breastfeeding are pain due to breastfeeding problems and (the perception of) insufficient milk supply.^5^ As we have detailed in our recent work,^6^ photonics offers many valuable opportunities for the development of more objective tools for lactation science and support. These tools can be employed to obtain unique insights into the physiology of the lactating breast,^7^^–^^9^ but also as an efficient method to quantify human milk composition.^10^^–^^12^ The latter is particularly important for monitoring the nutritional intake of premature newborns, who commonly require human milk fortification with additional nutrients to ensure optimal development. This patient group will likely benefit from a more personalized fortification approach, rather than the currently applied one-size-fits-all fortification that neglects the differences in patient specific milk intake and composition.^11^^,^^13^ To introduce and employ photonic methods for quantification of human milk composition, detailed knowledge on the interaction between human milk and light is required.

To evaluate milk composition in dairy industry, commercially available bovine milk analyzers estimate fat, protein, and lactose concentrations through near (NIR) and mid infrared (MIR) optical absorption spectroscopy.^14^ In the past decade, these analyzers have been adapted to quantify fat and protein concentrations in human milk with reasonable accuracy.^11^ As NIR and MIR human milk analyzers rely on relatively costly optical components, optical methods employing the visible wavelength range may offer a cost-effective alternative. Reducing costs may make milk analysis more widely available to support more mothers who need feedback on the nutritional intake of their infant.

In the visible wavelength range, the optical attenuation by milk is dominated by the scattering of fat globules (∼0.2 to 11  μm in diameter).^6^ In our recent work, we mapped the full set of optical properties of human milk and demonstrated significant correlations between milk fat concentration and the absorption coefficient $\mu_{a}$, scattering coefficient $\mu_{s}$, reduced scattering coefficient $\mu_{s}^{\prime }$, and backscattering coefficient $\mu_{b,\mathrm{NA}}$.^6^ Samples were homogenized through gently inverting by hand, to ensure that the fat globule size distribution was not affected by the sample preparation method. Building upon our previous work, this work aims to investigate the effect of other common and time-efficient sample preparation methods on the scattering properties of human milk: vortexing and sonication. This is essential knowledge for any future work on the optical investigation of human milk. Sonication is a crucial preparation step for NIR and MIR human milk analyzers, as it reduces the average size of the milk fat globules, and thereby reduces the cross talk from optical scattering to the measured absorption.^11^^,^^15^^,^^16^ Human milk sonication methods described in the literature are highly variable in terms of sonication power and duration.^11^^,^^15^^,^^16^

Next to fat globules, casein micelles (with radii around 100 nm) are the second most dominant scattering particles in human milk. Therefore, the second aim of this study is to also quantify their contribution to the scattering properties of human milk. Numerical results have previously shown that—due to their small size—casein micelles have a relatively small effect on $\mu_{s}$ (∼6%), whereas they do notably affect $\mu_{s}^{\prime }$ (∼43%, depending on fat concentration).^6^ Knowledge on the contribution of casein to the optical scattering properties of human milk is, therefore, relevant for both the quantification of fat content and the casein content itself.

In this work we quantified the scattering properties of human milk, by using our existing combination of spatially resolved diffuse reflectance spectroscopy (SR-DRS) and spectroscopic optical coherence tomography (sOCT).^6^ Human milk samples were homogenized with four different methods: (1) gently inverting the milk samples by hand, (2) vortexing, (3) sonication for 1 min, and (4) sonication for 5 min. The contribution of casein micelles was estimated by measuring the scattering properties before and after denaturation of the casein micelles. We explain our experimental results with Mie-theory, by modeling milk as a suspension of fat globules and casein micelles in whey.

## 2 Materials and Methods

### 2.1 Sample Collection Procedure

For this study, the DHMB (Dutch Human Milk Bank, Amsterdam, The Netherlands) provided mature human milk samples from five healthy donors with a lactation period between 2 and 8 months postpartum. All donors signed informed consent. The donors collected the milk samples between January 21, 2018, and March 15, 2018, at home with a breast milk pump, using standardized procedures. Immediately after collecting the milk samples in disposable bisphenol A-free bottles (Sterifeed, Medicare Colgate Ltd, Devon, England), the milk was stored in the donor’s home freezer at −18°C to −20°C. A carrier transported the milk to the DHMB for storage in a −20°C freezer, before the milk was transported to the University of Twente. There the milk was stored at −20°C for a maximum period of 11 months postexpression. Prior to the experiments, the samples were thawed in a 20°C water bath. Fat concentrations ranged from 21.5 to 41.3 g per kg milk, as we determined by a modified Mojonnier method.^6^^,^^17^

### 2.2 Preparation Methods

To study the dependency of the scattering properties of milk on the sample preparation method, we created five milk samples per donor by pipetting 5 mL of milk into five separate 10 mL falcon tubes. Hereafter, four samples were homogenized by four different methods: (1) gently inverting the sample by hand, (2) vortexing the sample for 10 s (Vortex-Genie 2, Scientific Industries, power 10), (3) sonication of the sample for 1 min (Sonifier 250, Branson, power 3, duty cycle 30%), and (4) sonication of the sample for 5 min with the same settings. During sonication, samples were cooled by placing the falcon tube into an ice bath. Performing these preparation methods resulted into four samples per donor.

The fifth milk sample was used to investigate the contribution of casein micelles to the scattering properties of milk. Hereto, we adapted the method from Stocker et al.^15^ to denature the casein micelles by adding an excess of 75  μl of 0.5-M ethylenediaminetetraacetic acid (EDTA) to the 5-ml milk sample. This denatures the casein micelles by destroying the calcium phosphate nanoclusters inside the casein micelles.^18^ After denaturation, casein micelle scattering becomes negligible.^15^ The samples containing EDTA were homogenized by gently inverting the sample by hand. Following all sample preparation methods described above, we prepared 5 samples per donor, yielding a total of 25 samples.

High concentrations of scattering particles may hinder the quantification of the scattering properties due to strong loss of signal in the SR-DRS setup and the contribution of multiply scattered light to the sOCT signal. Therefore, milk samples with an initial measured $\mu_{s}>6\;{\mathrm{mm}}^{-1}$ or $\mu_{s}^{\prime }>1\;{\mathrm{mm}}^{-1}$ at 550 nm were diluted with phosphate buffered saline until the results were below the mentioned values. Assuming concentration-independent single scattering events, the $\mu_{s}$ and $\mu_{s}^{\prime }$ of the original milk sample were retrieved by rescaling these scattering properties withthe dilution factor. All preparation methods were applied before dilution of the samples. All optical measurements in this work were performed in triplo. Hereto, each sample was measured three times while moving the sample in and out of the experimental setup in between consecutive measurements. Unless otherwise stated, all reported values are averages of the triplo measurement.

### 2.3 Bright-Field Microscopy

To study the effect of the preparation method on the fat globules in the milk, all samples were imaged with bright-field microscopy (EVOS FL Cell Imaging System, Thermo Fisher) at three different locations inside the sample. Hereafter, the fat globule size distributions were obtained by processing the images with MATLAB (R2017b, MathWorks) and its built-in “imfindcircles” function.

### 2.4 Experimental Estimation of the Scattering Properties

**Fig. 1:**
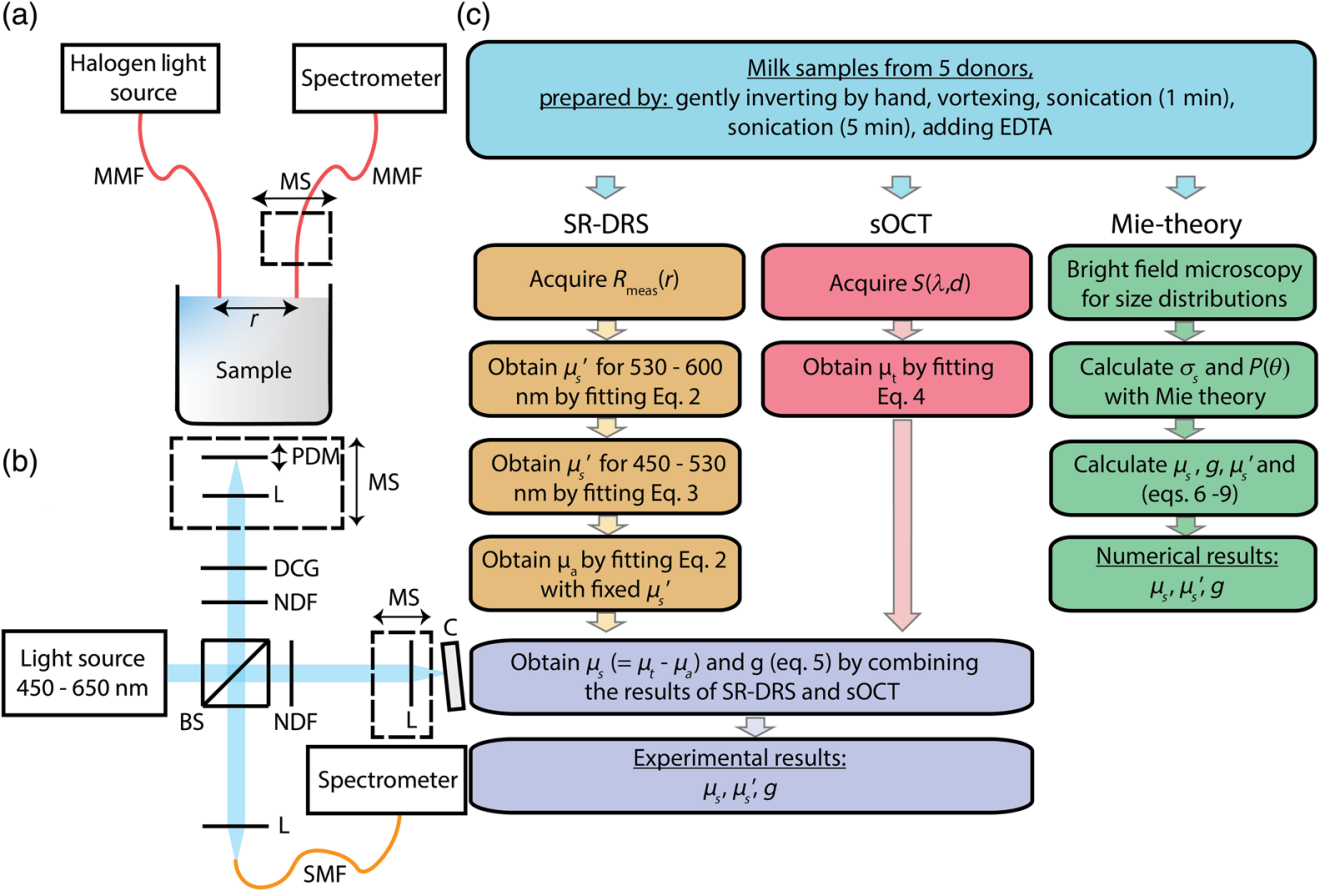
Schematic overview of the techniques used to estimate the scattering properties. (a) Illustration of the SR-DRS setup. Light from the illumination fiber diffuses through the sample, which was detected as a function of interfiber distance r by translation of the detection fiber. (b) Schematic overview of the sOCT setup. (c) Flowchart of the experimental and numerical methods in this study and the scattering properties they provide. MMF, multimode fiber; r, source–detector distance; MS, motorized stage; NDF, neutral density filters; L, lens; BS, beam splitter; DCG, dispersion compensation glass; PDM, piezodriven mirror; C, cuvette with sample; and SMF, single-mode fiber.

To experimentally estimate the scattering properties of all samples, we used the same methods as in our previous work.^6^ Hereto, we combined SR-DRS and sOCT to obtain the scattering properties ($\mu_{s}$, $\mu_{s}^{\prime }$, and g) of the milk samples. A schematic overview of the setups and a flowchart of the methods is given in Fig. 1, and a brief description of the methods is given below.

#### 2.4.1 Spatially resolved diffuse reflectance spectroscopy

The reduced scattering coefficient ($\mu_{s}^{\prime }$) of the milk samples was estimated by SR-DRS. We used a fiber-based home-built SR-DRS system that measured the diffuse reflectance as a function of interfiber distance r by translation of the detection fiber. The interfiber distance was varied from $r_{0}=2.2\;\mathrm{mm}$ to $r_{\text{end}}=4.7\;\mathrm{mm}$ in steps of 50  μm. The integration time was varied between samples and was set such that at $r_{0}$, 80% of the dynamic range of the spectrometer was filled. The dark-noise was obtained by a measurement without illumination.

The reduced scattering coefficient was obtained by adapting the model from Farrell et al.^19^ This model describes the diffuse reflectance as a function of the interfiber distance and the optical properties $\mu_{s}^{\prime }$ and $\mu_{a}$:

$$R_{\text{theory}}(r)=\frac{1}{4\pi }[\frac{z_{0}}{r_{1}^{2}}(\mu_{\mathrm{eff}}+\frac{1}{r_{1}})e^{-\mu_{\mathrm{eff}}r_{1}}+\frac{z_{0}+2z_{b}}{r_{2}^{2}}(\mu_{\mathrm{eff}}+\frac{1}{r_{2}})e^{-\mu_{\mathrm{eff}}r_{2}}].$$ (1)

With effective attenuation coefficient $\mu_{\mathrm{eff}}={\left[3\mu_{a}(\mu_{a}+\mu_{s}^{\prime })\right]}^{1/2}$, inverse total interaction coefficient $z_{0}={\left(\mu_{a}+\mu_{s}^{\prime }\right)}^{-1}$, $r_{1}={\left(Z_{0}^{2}+r^{2}\right)}^{1/2}$, $r_{2}={\left[{\left(z_{0}+2z_{b}\right)}^{2}\right]}^{1/2}$, and $z_{b}=(2/3){\left(\mu_{a}+\mu_{s}^{\prime }\right)}^{-1}$, under the conditions that $r>1/(\mu_{s}^{\prime }+\mu_{a})$ and $\mu_{s}^{\prime }\gg \mu_{a}$. For wavelengths smaller than 530 nm, absorption starts to play a more prominent role in the measured diffuse reflectance.^6^ Therefore, we follow our approach by Bosschaart et al.^20^ using a more robust two-step fitting approach over the wavelength range with (λ<530  nm) and (λ>530  nm) without absorption. Hereto, we first obtain $\mu_{s}^{\prime }$ for λ>530  nm by fitting Eq. (2) to the dark noise corrected $R_{\mathrm{meas}}(r)$ under the assumption of negligible absorption in this wavelength range:

$$R_{\mathrm{meas}}(r)=\beta R_{\text{theory}}(r),$$ (2)

with $\mu_{a}=0$ and fit parameters: scaling factor β and $\mu_{s}^{\prime }$. If the model validity condition ($r>1/\mu_{s}^{\prime }$ with $\mu_{a}=0$) was not satisfied, $r_{0}$ was increased until the condition was satisfied. Hereafter, $\mu_{s}^{\prime }$ was estimated for λ<530  nm by extrapolating a fitted power law to the results obtained from Eq. (2):

$$\mu_{s}^{\prime }=a\lambda^{-b},$$ (3)

with fit parameters: scaling factor a and scatter power b. Finally, we estimated $\mu_{a}$ by fitting Eq. (2) to $R_{\mathrm{meas}}(r)$ over the wavelength range of 450 to 600 nm, with $\mu_{s}^{\prime }$ fixed at the previously obtained values and fit parameters $\mu_{a}$ and β.

#### 2.4.2 Spectroscopic optical coherence tomography

Spectroscopic optical coherence tomography is an optical technique that allows for quantitativeand localized measurements of total attenuation, scattering, and absorption coefficients, as we have shown in our previous work for a wide range of samples ($\mu_{t}$ ranging from 0.15 to $34\;{\mathrm{mm}}^{-1}$).^21^^–^^24^ Here we used sOCT to estimate the scattering coefficient ($\mu_{s}$) of the milk samples.

We used a broadband Michelson-interferometer-based sOCT system [Fig. 1(b)], which measured the interference between the backscattered light from the sample and reference arm. A detailed description of our sOCT system is given by Veenstra et al.^24^ In short, we measured the backscattered spectra from the sample as a function of depth d. Our combined approach of focus tracking and zero-delay acquisition ensured that the measured attenuation of the OCT-signal with depth is affected only by the optical properties of the sample. The acquired spectra were short-time Fourier transformed with a spectral resolution of 5 nm resulting in a spatially and spectrally confined dataset of the backscattered intensity from the sample S(λ,d) with a spatial resolution ranging from 15  μm (at λ=450  nm) to 27  μm (at λ=600  nm). The lateral resolution of our system was 2.5  μm in air.

Under the assumption of single scattering, the attenuation spectrum ($\mu_{t}=\mu_{a}+\mu_{s}$) was obtained by fitting Lambert–Beer’s law to S(λ,d) for every wavelength in the range of 450 to 600 nm:

$$\ln\{{\left[S(\lambda ,d)-S_{\mathrm{bg}}(\lambda )\right]}^{2}\}=\ln[\alpha (\lambda )]-2\mu_{t}(\lambda )d,$$ (4)

with scaling factor α and $\mu_{t}$ as fit parameters. The background term $S_{\mathrm{bg}}(\lambda )$ was acquired by performing a measurement at a depth of 1 mm inside the sample, in which the OCT-signal had been completely attenuated. Hereafter, we quantified $\mu_{s}$ by correcting $\mu_{t}$ with the $\mu_{a}$ results from the SR-DRS measurement: $\mu_{s}=\mu_{t}-\mu_{a}$. Finally, the anisotropy g was estimated by combining the results of $\mu_{s}$ and $\mu_{s}^{\prime }$:

$$g=1-\frac{\mu_{s}^{\prime }}{\mu_{s}}.$$ (5)

#### 2.4.3 Methods accuracy

As shown in our previous work, the accuracy of sOCT for estimating $\mu_{t}$ is ∼10%.^22^ The accuracies of SR-DRS for estimating $\mu_{a}$ and $\mu_{s}^{\prime }$ are ∼15% and 10%, respectively.^20^ Since we obtained $\mu_{s}$ and g by combining the results from sOCT and SR-DRS, the inaccuracies of both methods propagate into the results for scattering properties. For human milk, $\mu_{a}$ is approximately 2 orders of magnitude smaller than $\mu_{s}$.^6^ Therefore, errors in the estimation of $\mu_{a}$ will be negligible for estimating $\mu_{s}$ through $\mu_{s}=\mu_{t}-\mu_{a}$. Since g is obtained from the ratio of $\mu_{s}$ and $\mu_{s}^{\prime }$, errors from both the SR-DRS and sOCT measurements propagate into g, resulting in an accuracy^6^ of 14% for 1−g.

### 2.5 Numerical Estimation of the Scattering Properties

To validate and explain our experimental results, Mie-theory was used to theoretically investigate the influence of sample preparation method on the scattering properties of the milk samples. With the fat globule size distributions from the bright-field microscopic measurements as an input, we modeled milk as a suspension of fat globules with variable radius $r_{\text{fat}}$ (refractive index^25^^,^^26^ $n_{\text{fat}}=1.46$) and monodisperse casein micelles in whey^27^ ($n_{\text{whey}}=1.345$). Since literature values for casein in human milk were unavailable, values of bovine milk were used:^28^^,^^29^ $n_{\text{casein}}=1.5$ and $r_{\text{casein}}=100\;\mathrm{nm}$. We used the MatScat software^30^^,^^31^ to individually calculate the scattering efficiencies $Q_{s}$ and phase functions P(θ) of casein micelles and fat globules—with $r_{\text{fat}}$ ranging from 0 to 5  μm—in whey at a wavelength of 550 nm. Subsequently, we calculated $\mu_{s}$ by multiplying $Q_{s}$ with the cross-sectional area $A=\pi r^{2}$ and the number of particles per volume $N=C/[(4/3)\pi r^{3}]$ with C the volume fraction of either fat or casein in the milk:

$$\mu_{s,\mathrm{Mie}}(r_{\text{fat}})=Q_{s,\text{fat}}(r_{\text{fat}})A_{\text{fat}}(r_{\text{fat}})N_{\text{fat}}(r_{\text{fat}})+Q_{s,\mathrm{cas}}A_{\mathrm{cas}}N_{\mathrm{cas}}.$$ (6)

All fat globule related parameters are a function of the measured fat globule size distribution $r_{\text{fat}}$, as Mie calculations were performed for the complete range of encountered fat globule radii. Since we investigated the influence of fat globule radius—and not fat concentration—on the scattering properties, we fixed the volume fractions at 0.325 (29 g fat per kg milk) for fat—which is the median fat volume fraction of the investigated milk samples in this study. The volume fraction of casein was fixed to a typical value^32^ of $1.68\times 10^{-3}$. To calculate the anisotropy $g(r_{\text{fat}})$, we first created a combined phase function by summing the unnormalized phase functions of fat globules and casein micelles:

$$P_{\mathrm{com}}(\theta ,r_{\text{fat}})=\frac{N_{\text{fat}}P_{\text{fat}}(\theta ,r_{\text{fat}})+N_{\mathrm{cas}}P_{\mathrm{cas}}(\theta )}{N_{\text{total}}},$$ (7)

after which $g(r_{\text{fat}})$ was calculated by

$$g_{\mathrm{Mie}}(r_{\text{fat}})=\frac{2\pi \int_{0}^{\pi }P_{\mathrm{com}}(\theta ,r_{\text{fat}})\cos\;\theta \;\sin\;\theta d\theta }{2\pi \int_{0}^{\pi }P_{\mathrm{com}}(\theta ,r_{\text{fat}})\sin\;\theta d\theta }.$$ (8)

Finally, $\mu_{s}^{\prime }$ ($r_{\text{fat}}$) can be obtained by combining the results for $\mu_{s}$ and g:

$$\mu_{s,\mathrm{Mie}}^{\prime }(r_{\mathrm{fat}})=\mu_{s,\mathrm{Mie}}(r_{\mathrm{fat}})[1-g_{\mathrm{Mie}}(r_{\mathrm{fat}})]$$ (9)

to compare the experimental results of the milk samples containing EDTA (i.e., with the casein micelles denaturated), all Mie calculations were also performed for $N_{\mathrm{cas}}=0$.

## 3 Results

### 3.1 Bright-Field Microscopy and Fat Globule Size Distributions

**Fig. 2:**
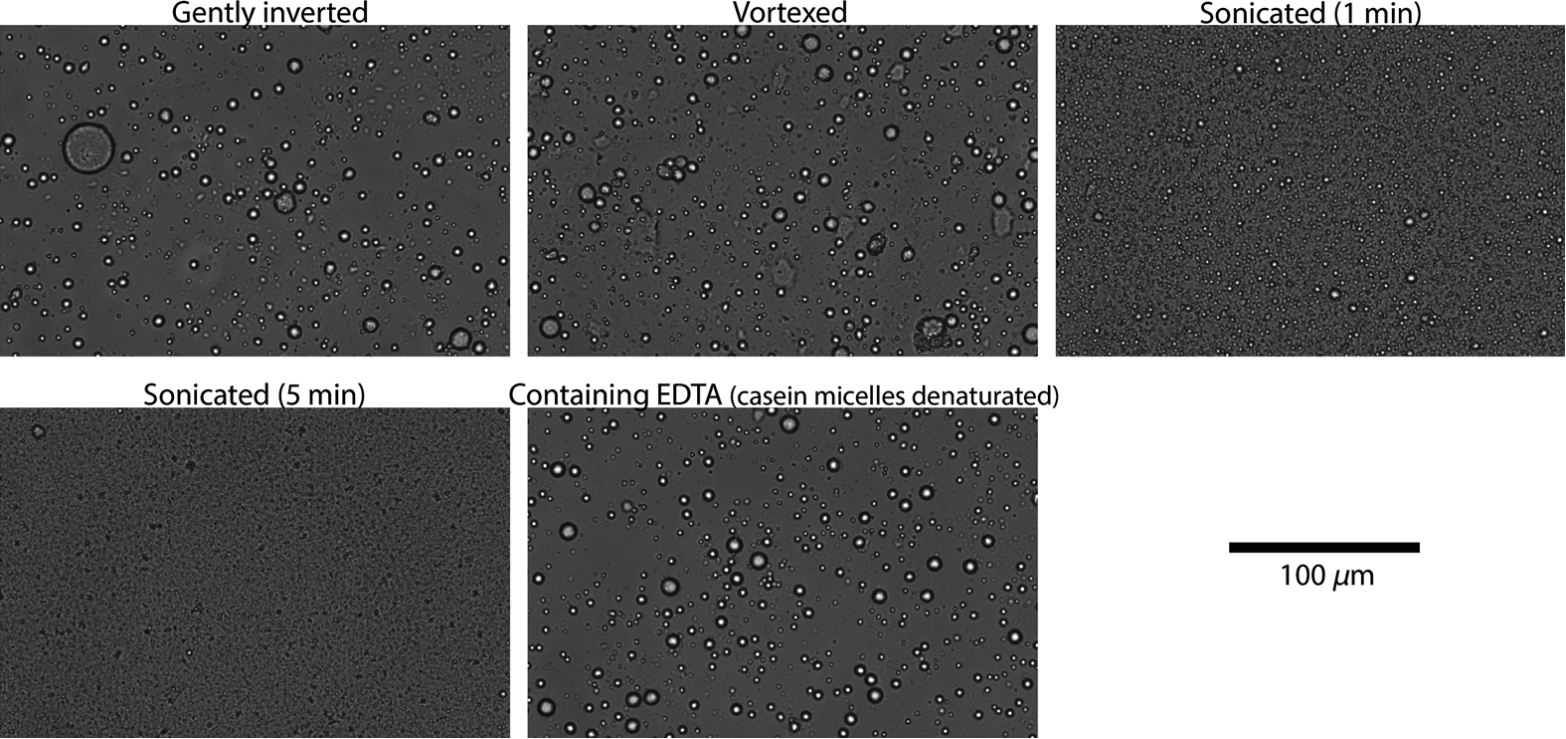
Typical bright-field microscopy images of samples prepared with the different methods used in this work.

**Fig. 3:**
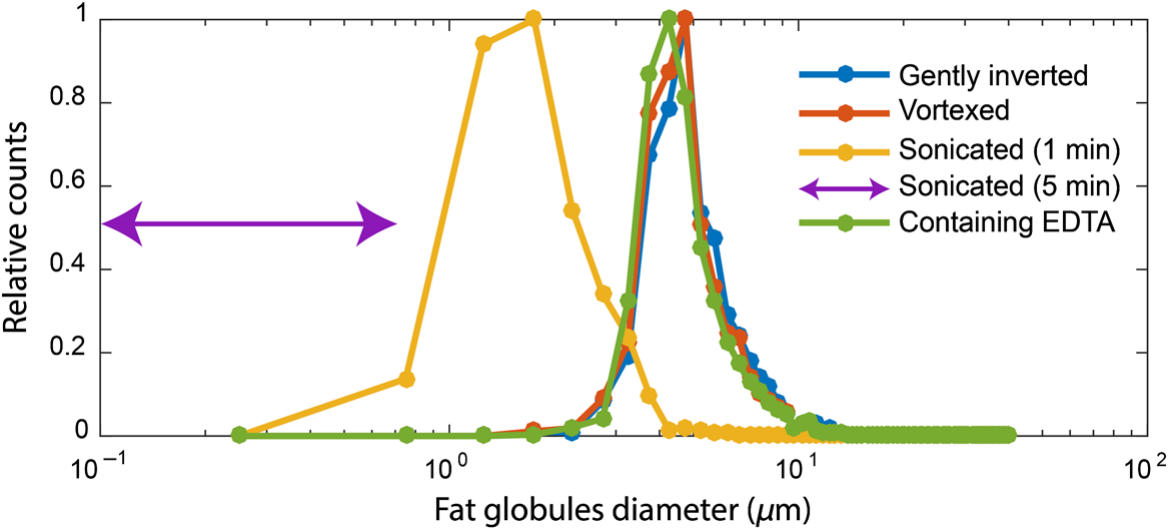
Fat globule size distributions for the different sample preparation methods used in this work. The average distributions of all participants are shown and lines serve as a guide to the eye. Due to the resolution limit of the bright-field microscope, the exact distribution of the samples that were sonicated for 5 min was not obtained. The arrows indicate that these samples, therefore, only contain particles <0.75  μm.

Figure 2 shows typical bright-field microscopy images for the four different sample homogenization methods and the addition of EDTA. The images demonstrate that homogenization by sonication reduces the size of the fat globules inside the milk samples. The fat globule size distributions (Fig. 3) show similar results for the gently inverted, vortexed, and EDTA samples with a maximum around 4.5  μm. The size distributions of the samples that were sonicated for 1 min have a maximum at a diameter of 1.75  μm. Due to the limited resolution (≈0.75  μm) of the bright-field microscope, no fat globule size distribution could be obtained for the samples that were sonicated for 5 min. The arrows in Fig. 3 indicate that these samples, therefore, only contain particles < 0.75  μm.

### 3.2 Optical Properties: Experimental Results

**Fig. 4:**
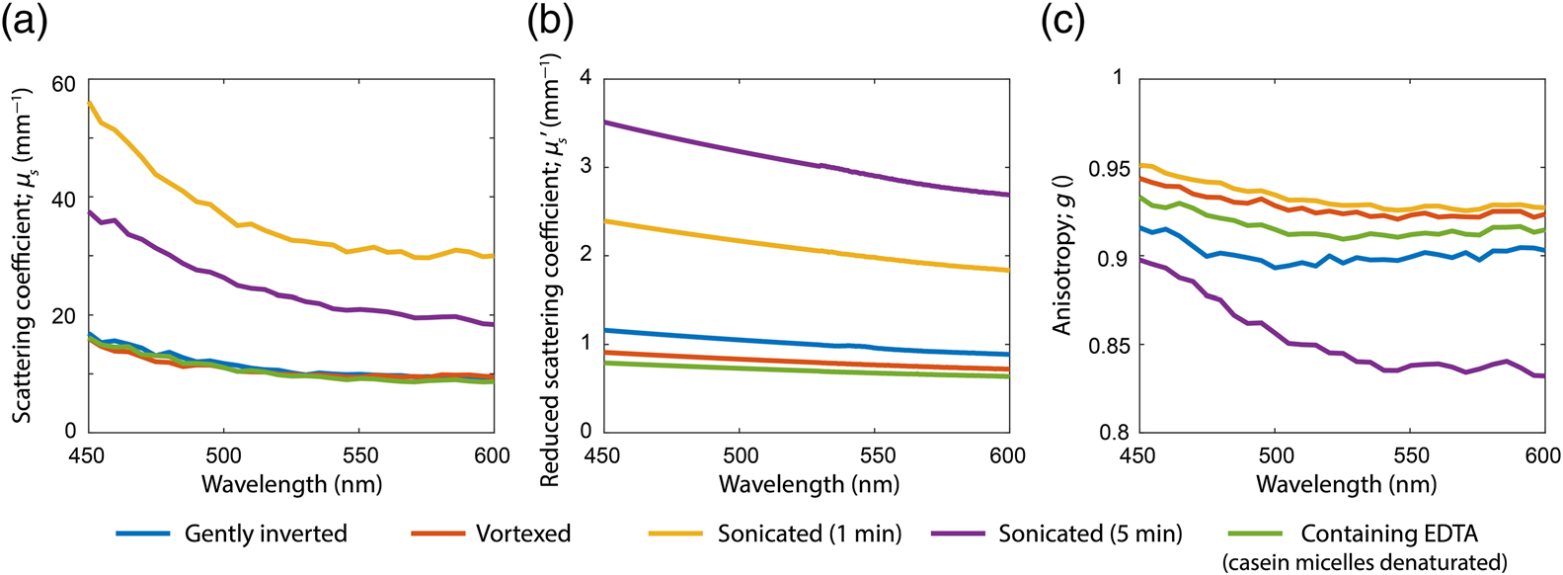
Experimentally derived scattering property spectra (medians of all donors) for all preparation methods. (a) Scattering coefficient $\mu_{s}$. (b) Reduced scattering coefficient $\mu_{s}^{\prime }$. (c) Anisotropy g.

The median values of the measured optical property spectra for all milk samples and processing methods are shown in Fig. 4 for the wavelength range of 450 to 600 nm. The median values and full range of the optical properties at 550 nm are listed in Table 1. All values mentioned in this section refer to the median values at 550 nm.

**Table 1:** Experimentally derived scattering properties at λ=550  nm. Results are shown as “median (full range)” for all samples.

Preparation method	$\mu_{s}$ (${\mathrm{mm}}^{-1}$)	$\mu_{s}^{\prime }$ (${\mathrm{mm}}^{-1}$)	g
Gently inverted	9.9 (5.1 to 11.2)	0.96 (0.64 to 2.1)	0.90 (0.81 to 0.91)
Vortexed	9.4 (6.0 to 10.8)	0.77 (0.40 to 1.2)	0.92 (0.89 to 0.93)
Sonicated (1 min)	30.0 (20.2 to 40.8)	2.0 (1.5 to 2.7)	0.93 (0.92 to 0.94)
Sonicated (5 min)	20.9 (16.3 to 23.7)	2.9 (1.9 to 4.5)	0.84 (0.81 to 0.90)
Containing EDTA	9.2 (5.4 to 10.7)	0.68 (0.47 to 0.99)	0.91 (0.88 to 0.93)

The results for $\mu_{s}$ were similar for the gently inverted, vortexed, and EDTA-containing samples. Relative to the gently inverted samples, 1 min of sonication resulted in a 203% increase in median $\mu_{s}$, whereas 5 min of sonication showed a 111% increase for $\mu_{s}$.

The vortexed and EDTA containing samples showed a 20% and 29% lower median $\mu_{s}^{\prime }$ than the gently inverted samples, respectively. Compared to gently inverting, 1 min of sonication resulted in a 108% increase for $\mu_{s}^{\prime }$, whereas 5 min of sonication resulted in an even stronger increase of 202%.

The vortexed samples showed higher anisotropy than the gently inverted samples. The samples that received 1 min of sonication showed the highest g with a value of 0.93, whereas the samples receiving 5 min of sonication with a value of 0.84 showed the lowest g. The samples that were vortexed (g=0.92) or containing EDTA (g=0.91) showed a slight increase in anisotropy compared to the gently inverted samples (g=0.90).

### 3.3 Optical Properties: Numerical Results

**Fig. 5:**
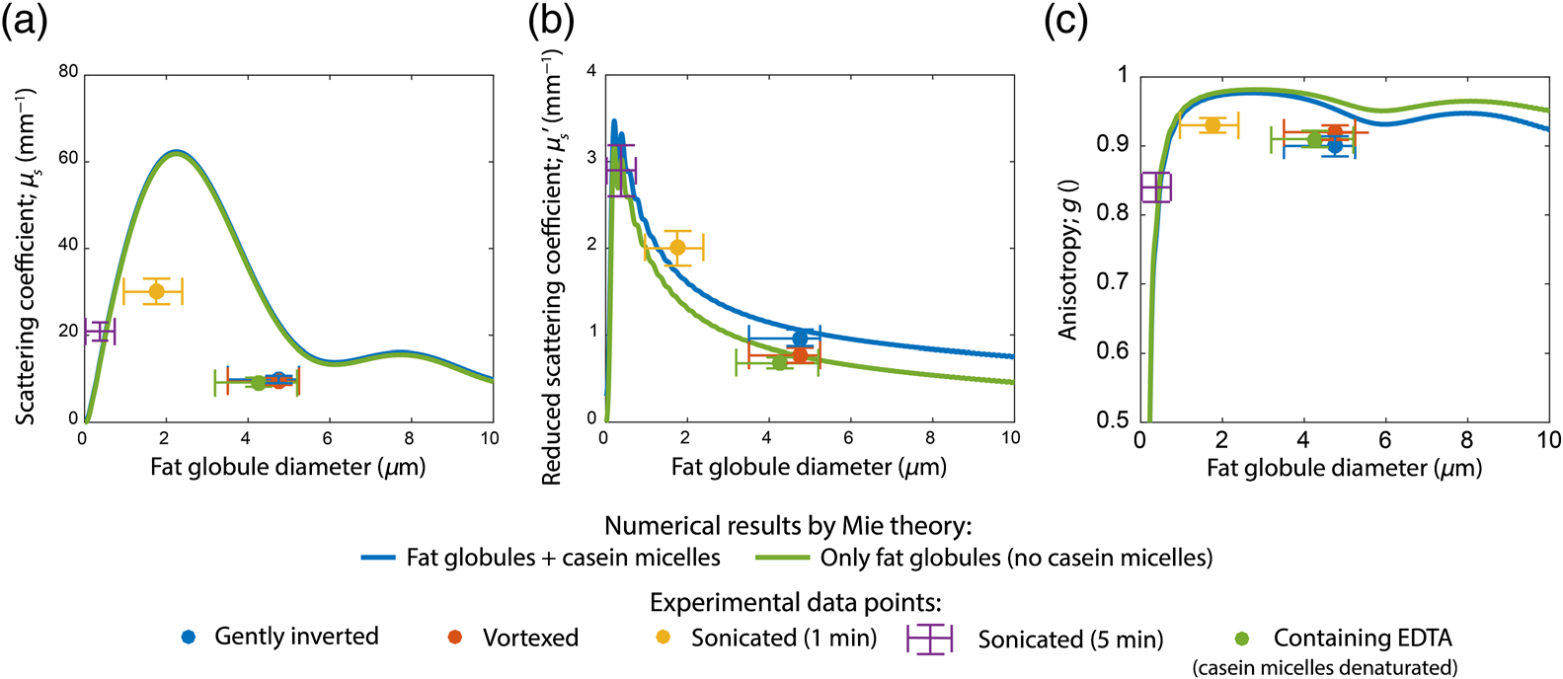
Numerical results from Mie-theory for the scattering properties of milk containing 32.5  ml/L of monodisperse fat globules both with and without casein micelles as a function of fat globule diameter. The concentration (1.68  ml/L) and particle radius (100 nm) of the casein micelles were constant for all Mie-calculations. Experimental results are shown for comparison; the horizontal position of the dots and horizontal error bars indicate the mode diameter and the full width half maximum of the corresponding fat globule size distribution respectively, whereas the vertical position of the dots and vertical error bars indicate the median experimental values and accuracies (as described in Sec. 2.4.3), respectively. Since a fat globule size distribution could not be obtained for the samples that were sonicated for 5 min, the horizontal error bars for this sample show the region of fat globule diameters lower than the microscope’s resolution. (a) Scattering coefficient $\mu_{s}$. (b) Reduced scattering coefficient $\mu_{s}^{\prime }$. (c) Anisotropy g.

Figure 5 shows the numerical results obtained by Mie-theory (solid lines) together with the median experimental results for comparison at λ=550  nm (data points). All scattering properties are presented as a function of fat globule diameter. Similar to our experimental results, Mie theory predicts a modest contribution of casein micelles to the scattering coefficient of $0.51\;{\mathrm{mm}}^{-1}$ [Fig. 5(a)]. Furthermore, Mie calculations show that for fat globules up to 2.2  μm, $\mu_{s}$ increases with fat globule size. Hereafter, $\mu_{s}$ decreases until a local minimum is reached at 6.1  μm. Although numerical results overestimate the experimentally obtained $\mu_{s}$ values, numerical and experimental results do show the same trend for $\mu_{s}$ as a function of fat globule diameter.

The Mie calculated $\mu_{s}^{\prime }$ increases with fat globule diameter up to a diameter of 210 nm, after which the $\mu_{s}^{\prime }$ starts to decrease with fat globule diameter [Fig. 5(b)]. Mie calculations show a casein micelle contribution of $0.30\;{\mathrm{mm}}^{-1}$ to the total $\mu_{s}^{\prime }$. The numerical and experimental results for $\mu_{s}^{\prime }$ are in good agreement.

The anisotropy of casein micelles was calculated to be 0.41. The Mie-calculations show a maximum of g=0.98 for milk consisting of fat globules with a diameter of 2.6  μm and a local minimum at g=0.93 for fat globules 5.9  μm in diameter [Fig. 5(c)]. The relative contribution of casein micelles is more pronounced for larger fat globules. Although the numerical results for g overestimate the experimental results, they do show the same trend with fat globule diameter.

## 4 Discussion

In this study, we measured the influence of common sample preparation methods and casein content on the scattering properties of human milk. As a validation, our experimental results were compared to Mie theory.

In general, we can conclude that sonication alters the scattering properties of the milk more strongly than vortexing or gentle inversion. Due to a reduction in fat globule size, both $\mu_{s}$ and $\mu_{s}^{\prime }$ increase with respect to the gently inverted samples. Although the increase in $\mu_{s}$ is strongest for the samples that underwent 1 min of sonication, the increase in $\mu_{s}^{\prime }$ is strongest for the samples that underwent 5 min of sonication (Fig. 4). This increase in $\mu_{s}$ and $\mu_{s}^{\prime }$ after sonication was also consistently observed within samples from individual donors. Qualitatively, this agrees well with our Mie calculations that show a maximum for $\mu_{s}$ around the fat globule size distribution of the samples that were sonicated for 1 min (Fig. 5). Also the Mie calculated values for g show a maximum around the fat globule size distribution of the samples that underwent 1 min of sonication, which explains the experimental results for g. Our experimental results for sonicated samples agree well with the study on bovine milk by Stocker et al.,^15^ who measured the same trends for $\mu_{s}$ and $\mu_{s}^{\prime }$ with sonication time.

Compared to gently inverting, we observed a 20% reduction in $\mu_{s}^{\prime }$ for the vortexed samples, whereas $\mu_{s}$ only showed a decrease of 5%. Since the fat globule size distributions of the gently inverted and vortexed samples are similar (Fig. 3), the decrease in $\mu_{s}^{\prime }$ cannot be ascribed to a difference in fat globule size. However, visual inspection of the vortexed milk samples revealed the presence of aggregates, which can be formed by the flocculation or coalescence of colliding fat globules during the vortexing process.^33^ As SR-DRS probes a much larger volume compared to sOCT, it is more likely that these aggregates will be present in the SR-DRS probing volume. As a consequence, they will have a larger effect on the measurement of $\mu_{s}^{\prime }$ compared to that of $\mu_{s}$.

Although the trends between the experimental and numerical results for the different sample preparation methods are similar, our Mie calculations tend to overestimate the experimental results for $\mu_{s}$ and g in absolute terms. Potential causes are: (I) Mie theory assumes scattering by homogeneous spheres. This is a simplified model of fat globules as they consist of a lipid core surrounded by a phospholipid membrane.^34^ (II) We used the refractive index of fat globules in bovine milk as input parameter for the Mie calculations. The currently unreported refractive index of fat globules in human milk may be different. (III) The refractive index of whey is assumed to be constant, whereas variations in the composition of the whey will affect the whey’s refractive index. For skimmed bovine milk, the refractive index has been reported^35^ to range between 1.345 and 1.348. (IV) Inaccuracies in the chemically derived fat concentrations,^6^ which were used as an input parameter for Mie calculations. (V) The use of bright-light microscopy can induce inaccuracies in the fat globule size distributions that were used to compare our experimental results with the Mie calculations (Fig. 5). In bright-field microscopy, a trade-off exists between the visibility of smaller and larger fat globules due to the limited depth of focus of the imaging plane. Out of focus fat globules will appear larger in the microscopic image than their actual size, which induces a right shift of the resulting fat globule size distribution. As we used a bin size of 0.5  μm to create the size distributions, this effect will be most prominent for the fat globule size distributions that contain the smallest particles.

Regarding the contribution of casein micelles to the scattering properties of human milk, the experimental and numerical results agree well on a quantitative level for $\mu_{s}^{\prime }$. At λ=550  nm, the absolute values for $\mu_{s}^{\prime }$ of the gently inverted samples and the samples containing EDTA (casein micelles denaturated) are in good agreement with the Mie calculated values (Fig. 5), indicating a casein contribution of $0.3\;{\mathrm{mm}}^{-1}$ to $\mu_{s}^{\prime }$. This implies that the presumed casein concentration of 1.68  mL/L for the Mie calculations is close to the concentration in the investigated milk samples. This finding is supported by the fact that casein concentrations in human milk show only minor biological variability compared to its fat concentration.^32^ For bovine milk, the reported contribution of casein micelles to $\mu_{s}^{\prime }$ is higher,^15^ with $∼1.75\;{\mathrm{mm}}^{-1}$. This difference can be explained by the higher concentrations of casein in bovine milk compared to human milk.^32^^,^^36^ Due to the small size of casein micelles compared to the fat globules in human milk, both our experimental results and Mie calculations show a negligible influence of casein on $\mu_{s}$.

The measurements in this work were performed on samples that have been frozen during storage. Although milk samples are ideally measured directly after expression from the breast, such samples were not available during this study. As a consequence, we were unable to investigate the influence of freezing, storing, and thawing of milk samples on their scattering properties. Nevertheless, there are indications that freeze–thaw procedures may affect fat globule size.^37^ Future research is necessary to reveal the influence of also these processing steps on the optical scattering properties of human milk.

This work shows that the scattering properties of human milk strongly depend on the preparation method, which is essential knowledge for the optical investigation of human milk, including the optical quantification of human milk composition. As sonication is a standard sample preparation procedure for NIR and MIR human milk analyzers, any future work on combining these methods with visible light techniques will have to take into account the effect of sonication on the optical scattering properties. As vortexing human milk samples may introduce the presence of fat globule aggregates that affect the measurement of $\mu_{s}^{\prime }$, the preferred method for human milk sample preparation will be gently inverting. Another outcome of this work is the quantitative contribution of casein micelles to the scattering properties of human milk. Although casein concentrations are relatively stable for mature human milk, concentrations may be different and show more biological variability in colostrums.^38^ As such, these findings are important for both the optical quantification of fat content as well as the casein content itself.

## 5 Conclusions

In this study, we demonstrated experimentally and numerically that the scattering properties of human milk are strongly affected by common sample preparation methods. Sonication most strongly influenced all scattering properties of human milk due to a reduction of fat globule size but also vortexing affected the reduced scattering coefficient $\mu_{s}^{\prime }$. For homogenization purposes, gently inverting should, therefore, be the preferred method. The contribution of casein micelles to the scattering properties of human milk is relatively low for $\mu_{s}$ and g (7.1% and 1.1% at 550 nm, respectively), but considerably larger for $\mu_{s}^{\prime }$ ($29\%\;{\mathrm{mm}}^{-1}$ at 550 nm).
